# Complete Genome Sequence of a Novel Recombinant *Citrus Tristeza Virus*, a Resistance-Breaking Isolate from Uruguay

**DOI:** 10.1128/genomeA.00442-18

**Published:** 2018-05-31

**Authors:** María José Benítez-Galeano, Thomas Vallet, Lucía Carrau, Lester Hernández-Rodríguez, Ana Bertalmío, Fernando Rivas, Leticia Rubio, Diego Maeso, Marco Vignuzzi, Gonzalo Moratorio, Rodney Colina

**Affiliations:** a Laboratory of Molecular Virology, University of the Republic, Salto, Uruguay; b Institut Pasteur, Viral Populations and Pathogenesis, Centre National de la Recherche Scientifique UMR 3569, Paris, France; c National Institute of Agricultural Research, Salto, Uruguay

## Abstract

We report here the complete genome sequence of a Citrus tristeza virus (CTV) from Uruguay, sequenced by using Illumina and Sanger sequencing technology. This CTV DSST-17 genome clustered within genotype resistance breaking (RB) and presents two recombination events.

## GENOME ANNOUNCEMENT

Citrus crops are among the most important commercial fruit crops worldwide. Citrus tristeza virus (CTV) (Closterovirus, Closteroviridae) is one of the most destructive pathogens that affects citrus trees around the world and has been responsible for the loss of over 100 million trees in the past 70 years (1). Depending on the viral strain and on the species or scion-rootstock combination, CTV may cause three distinct host reactions, named seedling yellows, quick decline, and stem pitting, of which the last two are significant problems for citrus cultivation (2).

The CTV genome, the largest plant virus reported so far, is a single-stranded positive-sense RNA molecule of approximately 19.3 kb in length, containing 12 open reading frames (ORFs) that encode at least 19 proteins (3). Genetic studies of different strains of CTV revealed the existence of seven distinct genetic lineages or genotypes worldwide, known as VT, T3, T30, T36, T68, resistance breaking (RB), and NC (4, 5). The RB genotype, described for the first time in New Zealand by Dawson and Mooney, is the only CTV-infecting genotype capable of overcoming the trifoliate-rootstock resistance due to the ability of replication and systemic movement throughout Poncirus trifoliata (6). Last year, Hernández-Rodríguez and coworkers reported a New Hall sweet orange tree infected with the CTV-RB genotype in Uruguay, but only partial sequences were available (7).

In the present study, subisolate DSST-17, obtained by single aphid transmission from a field sample collected in 2014 in Salto, Uruguay, from a Navelina sweet orange, was subjected to Illumina sequencing technology. Total RNA was extracted using the RNeasy plant minikit (Qiagen) and submitted to RNA library preparation with a NEBNext Ultra II RNA library prep kit (Illumina). The library was sequenced using the NextSeq 500 system platform (Illumina). Reads were trimmed (quality limit 0.02; Phred score ≥ 30) and assembled with CLC Genomics Workbench version 11. After trimming, reads with an average length of 150 nucleotides (nt) were used to generate through *de novo* assembly a long contig of 19,269 nt. The complete genome obtained was compared with all available full genomes of the GenBank database using MEGA 6 (8). Phylogenetic analysis grouped the DSST-17 isolate within the RB genotype with a genome nucleotide identity ranging from 94.1% to 99.6%. Strikingly, the highest similarity was with isolate B390-5 (GenBank accession number KU883265) from South Africa, which has weather conditions similar to those of Uruguay. A recombination analysis using the Recombination Detection program version 4 and SimPlot program version 3.5.1 was performed (9, 10). The analysis revealed that isolate DSST-17 is a recombinant genome with at least two recombination events. For the first recombinant region, a T36-like fragment goes from positions 1 to 3616. The second event seems to be with an NC-like isolate (HA16-5) from base 14586 to the end of the genome. In addition, PCR amplification and Sanger sequencing were performed to confirm these recombination events. As far as we know, this is the first report from South America of a complete genome of CTV belonging to the RB genotype.

### Accession number(s).

The genomic sequence for isolate CTV DSST-17 was deposited in GenBank under accession number MH186146.
